# Diet shifts provoke complex and variable changes in the metabolic networks of the ruminal microbiome

**DOI:** 10.1186/s40168-017-0274-6

**Published:** 2017-06-08

**Authors:** Sara M. Wolff, Melinda J. Ellison, Yue Hao, Rebecca R. Cockrum, Kathy J. Austin, Michael Baraboo, Katherine Burch, Hyuk Jin Lee, Taylor Maurer, Rocky Patil, Andrea Ravelo, Tasia M. Taxis, Huan Truong, William R. Lamberson, Kristi M. Cammack, Gavin C. Conant

**Affiliations:** 1 Division of Animal Sciences, University of Missouri-Columbia, Columbia, MO USA; 2 Informatics Institute, University of Missouri-Columbia, Columbia, MO USA; 3 Division of Biological Sciences, University of Missouri-Columbia, Columbia, MO USA; 4 Department of Animal and Veterinary Science, University of Idaho, Moscow, ID USA; 5 Department of Dairy Science, Virginia Polytechnic Institute and State University, Blacksburg, VA USA; 6 Department of Animal Science, University of Wyoming, Laramie, WY USA; 7 Department of Computer Science, Truman State University, Kirksville, MO USA; 8 Department of Psychology, Truman State University, Kirksville, MO USA; 9 Department of Biology, Kenyon College, Gambier, Ohio USA; 10 National Animal Disease Center, ARS, USDA, Ames, IA USA; 11 Department of Animal Sciences, South Dakota State University, Brookings, SD USA; 12 Program in Genetics, North Carolina State University, Raleigh, NC USA; 13 Bioinformatics Research Center, North Carolina State University, Raleigh, NC USA; 14 Department of Biological Sciences, North Carolina State University, Raleigh, NC USA

**Keywords:** metagenomics, vertebrate microbiome, metabolic network

## Abstract

**Background:**

Grazing mammals rely on their ruminal microbial symbionts to convert plant structural biomass into metabolites they can assimilate. To explore how this complex metabolic system adapts to the host animal’s diet, we inferred a microbiome-level metabolic network from shotgun metagenomic data.

**Results:**

Using comparative genomics, we then linked this microbial network to that of the host animal using a set of interface metabolites likely to be transferred to the host. When the host sheep were fed a grain-based diet, the induced microbial metabolic network showed several critical differences from those seen on the evolved forage-based diet. Grain-based (e.g., concentrate) diets tend to be dominated by a smaller set of reactions that employ metabolites that are nearer in network space to the host’s metabolism. In addition, these reactions are more central in the network and employ substrates with shorter carbon backbones. Despite this apparent lower complexity, the concentrate-associated metabolic networks are actually more dissimilar from each other than are those of forage-fed animals. Because both groups of animals were initially fed on a forage diet, we propose that the diet switch drove the appearance of a number of different microbial networks, including a degenerate network characterized by an inefficient use of dietary nutrients. We used network simulations to show that such disparate networks are not an unexpected result of a diet shift.

**Conclusion:**

We argue that network approaches, particularly those that link the microbial network with that of the host, illuminate aspects of the structure of the microbiome not seen from a strictly taxonomic perspective. In particular, different diets induce predictable and significant differences in the enzymes used by the microbiome. Nonetheless, there are clearly a number of microbiomes of differing structure that show similar functional properties. Changes such as a diet shift uncover more of this type of diversity.

**Electronic supplementary material:**

The online version of this article (doi:10.1186/s40168-017-0274-6) contains supplementary material, which is available to authorized users.

## Background

Ruminant mammals are remarkable for their ability to subsist off of plant structural compounds such as cellulose that are at once hugely abundant on the landscape and yet metabolically inaccessible to most animals [1–4]. Aside from its obvious economic implications in animal husbandry [5, 6] and links to improving human and animal health [7–9], this metabolic capacity is potentially of great importance for applications in biotechnology, from that of using plant matter to generate low-carbon footprint fuels [10–12] to less expected ones, such as environmental remediation [13].

At the same time, the ruminal microbial ecosystem is a useful model of how ecosystems develop and operate [3] because it is contained and yet susceptible to experimental manipulation, for instance by shifting the diet of the host organism or by antibiotic treatment [14]. The first step in studying such an ecosystem is to catalog its players, namely to explore the species present and their taxonomic relationships. There is a long tradition of such work in ruminal microbiology, starting with culture-based studies [1, 15, 16], and continuing to gel [5] and sequencing-based approaches [17–21]. These taxonomic studies have reinforced just how unusual the ruminal microbial ecosystem is: it forms an outlier not merely to the microbiomes of other animals but even to other more general terrestrial and marine microbial ecosystems [22].

Nonetheless, while powerful, a taxonomic approach to microbial ecology has certain limitations. For instance, microbial ecosystems can show taxonomic differences that mask similarities at the metabolic or functional level [7, 23–28]. Perhaps more seriously, the observations generated by taxonomic studies are generally correlative or based on other types of statistical association (such as principal component analysis; PCA). In particular, generating hypotheses about the global structure of the microbiome is currently challenging. For example, it is intuitive to expect that a diet that consists of compounds similar to those used by the host’s metabolic network would induce a metagenomic metabolic network more related to that of the host than would a more complex diet with difficult-to-digest compounds. But this idea would be quite difficult to test using only taxonomic identifiers, even in the unlikely event that the metabolic capacities of those organisms was completely known. One solution to these difficulties is to extend the analysis of the microbiome to include an analysis of its full gene complement [10, 11, 29, 30].

**Fig. 1:**
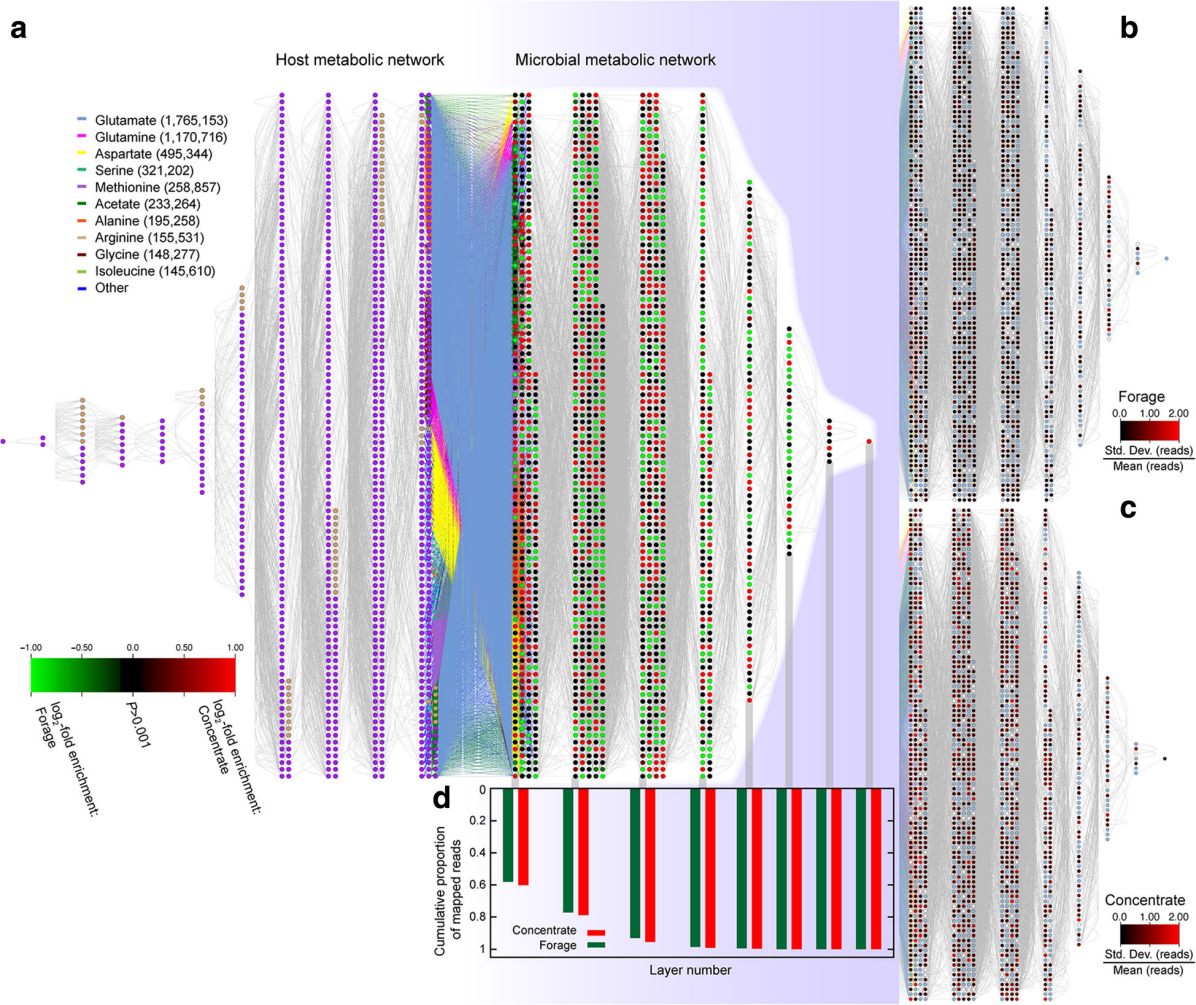
Merged host/microbe metabolic network. **a** Each node (circle) is a reaction in the host genome (*left*) or microbial metagenomes (*right*). Host nodes are colored purple if derived from an orthology association between sheep and humans, tan if from an ovine/bovine relationship or blue for the case of the added buyrate-employing pseudo-reaction (Materials and Methods). Edges are shared metabolites (network *N*_*50*_). In the center are nodes employing 23 potentially shared compounds between the host and microbes (set VFA + AA; Materials and Methods): the 10 most frequent metabolites (by microbe read count to their respective reactions) are individually colored. All nodes are organized by their distance from the other subnetwork: hence nodes employing an interface metabolite are at the center with a distance of 0. Microbial nodes are colored based on the normalized log_2_-fold difference in read count between the two diets (green: overabundant in the FORG diet; red: overabundant in CONC). Nodes whose normalized read counts did not differ significantly between the diets are shown in black (Materials and Methods). **b** The right half of part **a**, recolored based on the normalized animal-to-animal variance in read count for the FORG animals (see scale bar). **c** Same as for **b** but for the CONC animals. **d** Histogram giving the cumulative proportion of the total mapped reads for the two diets (*green*: FORG; red: CONC) at each level of the network. Note that the FORG animals have proportionally more reads mapped to more distant layers of the network

The power of such gene-centric analyses can be considerably enhanced with tools from network and systems biology that can provide linkages between the different genes [31–34]. We have previously applied a metabolic network approach [32, 33] to the ruminal microbiome of cattle [26]. In this approach, genes are annotated to biochemical reactions using a database such as MetaCyc [35]: these reactions are then connected to each other via shared metabolites (Fig. 1). In addition to further illustrating the potential dangers of relying only on taxonomic signals to understand the structure of these microbial communities [7, 23, 24, 36], our analyses suggested that the metabolic interface between these microbes and the vertebrate host provides structure to this community metabolic network [26, 32].

Here we explore how a shift in host diet alters the metabolic capacity of the sheep rumen microbiome. We previously showed that there was a significant shift in taxonomic composition that resulted from such a diet change [18]. However, given our observations in cattle [26], it remains possible that these taxonomic differences were not fully reflected in the metabolic network. Indeed, we hypothesized that we would see conservation in the parts of the metabolic network that interact directly with host metabolism, with larger changes in other regions of the network.

Instead, we found that a change to a carbohydrate diet resulted in a shifting of the microbial network closer to that of the host, coupled to a reduction in complexity of that microbial network. These shifts varied considerably between animals, and, in our simulations of metabolic network changes, we found that a diet change can indeed induce this type of inter-animal variation.

## Methods

### Animal Use and Rumen Sample Collection

Rumen fluid was sampled from 16 wethers of Rambouillet, Hampshire, and Suffolk breeds. After 24 days on a shared diet of primarily hay supplemented by maize, eight animals were randomly assigned to a pelleted, concentrate-based diet (CONC: main component maize), while the remaining animals were fed a pelleted, forage-based diet (FORG: main component alfalfa). Animals were acclimated to the new diets over 25 days, after which a 49 day feed trial was completed on the new diets. Rumen fluid samples were collected via oral lavage and frozen at -80 ° C. Full details of the feeding experiment are given in [18].

### DNA Extraction & Library Preparation

Thawed 1 ml rumen samples plus sterilized zirconia (0.3 g of 0.1 mm) and silicon (0.1 g of 0.5 mm) beads and 1 ml of lysis buffer were homogenized with a Mini-Beadbeater-8 (three minutes), incubated at 70 °C (15 minutes + mixing every 5 minutes), and centrifuged at 4 °C (16,000 g for 5 minutes). The resulting supernatant was transferred to new tubes and fresh lysis buffer was added. This process was repeated a second time, and supernatants pooled. The QIAamp DNA Stool Mini Kit (Qiagen, Santa Clarita, CA, USA) was then used to precipitate nucleic acids and to remove RNA and proteins.

Genomic libraries were constructed from these 16 DNA samples using Illumina’s Illumina TruSeq DNA PCR-free Library Prep kit according to the manufacturer’s recommended protocol [18]. Genomic DNA was sheared into fragments using Diagenode BioRuptor. The resulting 3′ and 5′ overhangs were removed, adenosine nucleotide added to 3′ ends, and Illumina adapters ligated. Fragments of 420 base pairs (bp) were size selected as described in the Illumina protocol. Qubit assays were used to quantify each library, and fragment size confirmed by the Agilent BioAnalyzer High Sensitivity DNA assay.

### Illumina Sequencing

Each sample was sequenced on an Illumina HiSeq 2000 at four samples per flowcell lane, resulting in 100 base-pair, paired end sequences, with mean insert size of 309 bp. Raw sequence reads are available from NCBI’s short read archive (Project SRP028527). We truncated each read after the first run of three bases with phred quality score less than 15 [37]. If either read had an average quality score less than 25 or was shorter than 85 bases, that read pair was omitted. After filtering, 96 gigabases of sequence remained (Table 1).

**Table 1:** Read Statistics by Animal

ID	Diet	Total reads^a^	Reads with valid paired ORFs^b^	# (%) reads that hit to nodes^c^	# of reads hitting multiple nodes	# (%) reads hit to sheep genome^d^	#OTU found^e^
1003	FORG	16,779,099	14,521,805	271,571 (1.87%)	220,794	58,830 (0.35%)	109
1009	FORG	35,930,923	30,282,660	761,728 (2.52%)	548,931	1,855,867 (5.17%)	161
1127	FORG	41,120,479	35,920,407	570,177 (1.59%)	459,714	63,121 (0.15%)	137
1208	FORG	44,823,544	39,038,632	1,012,146 (2.59%)	790,442	18,155 (0.04%)	140
1248	FORG	22,698,997	19,869,280	673,066 (3.39%)	441,001	2,804 (0.01%)	127
1366	FORG	18,124,115	14,952,153	396,240 (2.65%)	275,291	42,582 (0.23%)	119
1397	FORG	32,221,706	28,176,562	657,645 (2.33%)	486,817	62,527 (0.19%)	137
7505	FORG	47,234,706	38,189,761	651,061 (1.70%)	478,999	1,175,563 (2.49%)	177
1026	CONC	29,835,213	24,169,312	642,601 (2.66%)	780,456	7,564 (0.03%)	108
1101	CONC	54,927,600	47,578,081	1,008,933 (2.12%)	1,059,980	44,188 (0.08%)	142
1111	CONC	26,710,771	23,016,865	362,506 (1.57%)	486,293	167,325 (0.63%)	137
1220	CONC	7,800,938	6,274,376	109,479 (1.74%)	126,946	1,226 (0.02%)	75
1239	CONC	42,216,924	36,551,070	751,236 (2.06%)	775,268	24,083 (0.06%)	138
1348	CONC	13,577,697	11,843,596	245,424 (2.07%)	246,823	12,233 (0.09%)	102
1396	CONC	18,274,753	15,860,653	278,963 (1.76%)	339,002	7,330 (0.04%)	124
7429	CONC	30,236,139	26,315,246	553,079 (2.10%)	537,174	65,498 (0.22%)	135

^a^Total paired reads sequenced prior to quality filtering

^b^Total number of paired reads passing read quality filtering and having a sufficiently long ORF in both members (Materials and Methods)

^c^Number and percent of valid reads (previous column) that were mapped to nodes according to our criteria (Materials and Methods)

^d^Number and percent of total reads that mapped to the sheep genome at 80% percent identity

^e^Number of distinct OTUs identified previously in these sequences [18]

### OTU Analysis

As described [18], the reads were compared to the Ribosomal Database Project [38] using Bowtie [39] to identify fragments of 16S rDNA genes. We retained hits that matched sequences in the database at ≥97%. OTUs were defined by single-linkage clustering [40] in the original database: read pairs that mapped to exactly one such OTU were considered instances of that OTU. The result was the identification of a total of 349 OTUs [18].

### Metabolic network inference from MetaCyc

MetaCyc includes more than 2000 microbial metabolic networks [35], with enzyme sequences annotated to reactions and each reaction annotated with substrates and products. Using these reactions and their respective metabolites, we inferred a global metabolic network for all bacteria and archaea in MetaCyc, merging reactions with identical metabolites, to yield a total of 6140 reactions/nodes [26]. We then linked that network to that of the host animal by using comparative genomics to infer a sheep metabolic network. For both networks, nodes were defined as metabolic reactions (catalyzed by enzymes): edges connect nodes that have a metabolite in common (lines and circles, respectively, in Fig. 1). Isolated reactions were omitted.

As there is no ovine network in MetaCyc, we merged the human metabolic network with the bovine reactions not found in humans and used enzyme orthology to infer whether each enzyme in this merged network was found in the sheep genome. As described in several previous contributions [41–44], we started with Ensembl release 75 [45] of the human, ovine and bovine genomes and used our synteny-based orthology inference package to infer in human-ovine and ovine-bovine orthologs. We found 15562 human-ovine and 15479 bovine-ovine ortholog pairs. To allow for the known ability of the host to incorporate microbially-produced butyrate, we added, to the ovine orthologs of 1834 human reactions and 154 bovine reactions, a host-based pseudo-reaction converting butyrate to butyryl-CoA, giving a total of 1989 nodes in the inferred ovine metabolic network.

For both the microbial and host networks, there are metabolites, known as “currency metabolites,” that occur in so many reactions that they give rise to spurious linkages between unrelated parts of the metabolic network. For instance, the presence of water or a proton in the list of reactants for two reactions should not be taken to indicate that the two reactions are associated. Because there is no universal definition of a currency metabolite [44], we ran all analyses three times using currency cutoffs of 25, 50 and 100: in other words, we did not create edges for metabolites occurring in 25 or more, 50 or more or 100 or more reactions [46]. We refer to these networks as N_25_, N_50_ and N_100_ (261, 206 and 174 currency metabolites removed, respectively).

### Mapping translated reads to the microbial metabolic network

To identify MetaCyc reactions present in the shotgun sequences, we first translated our paired reads (having untrimmed lengths of 200 base pairs in total) in all six open reading frames (ORFs). The resulting paired amino acid sequences were discarded unless both had a translated ORF longer than 29 residues. We note that analyses by Carr and Borenstein [47] have suggested that the accuracy of using read mapping to infer function improves considerably when using 200 base pair reads relative to using 100 base pair reads but increases much more slowly thereafter. Using the SeqAn library [48], we searched for translated reads that, after Smith-Waterman local alignment [49], matched database sequences at a) two identical seven residue “words,” b) >80% amino acid identity and c) over 80% of the metagenomic ORF. Reads mapping to multiple database sequences catalyzing the same reactions, or to sequences catalyzing a subset of those reactions, were assigned to their respective nodes. Translated reads that mapped to multiple non-related reactions or where the forward and reverse reads mapped to differing enzymes were discarded.

To validate this mapping approach, we analyzed a random set of 20,000 “pseudo-reads” created from the original MetaCyc enzyme database. These simulated reads were drawn in pairs from enzyme sequences and each had a length of 30 amino acids, with a simulated unsequenced insert of 70 amino acids. Using the same mapping software, we aligned them to the database (omitting the sequence the read pair was simulated from) and computed the ratio of uniquely mapping to multiplying mapping reads for these data (Additional file 1: Figure S1A).

The choice of a threshold of 80% percent identity is supported by three observations. First, in our analyses of the trends in total matched reads and in the percent of reads matching to multiple reactions across a range of this cutoff in percentage identity, we found that the proportion of reads having valid hits to the enzyme database began to plateau near this threshold, while the proportion of reads mapping to multiple reactions was still increasing (Additional file 1: Figure S1A). Second, we found that at the very high cutoff of 95% amino acid identity, substantially more reads mapped from samples drawn from animals fed a forage diet than matched with samples drawn from concentrate-fed animals (0.45% and 0.17% of reads mapping, respectively). This bias in mapping effectiveness yielded a higher mean layer number for the concentrate animals (see *Results*) in contrast to the lower mean layer number seen at cutoffs of 90% or 80%, where the mapping efficiency was more balanced between the two diets. Third and finally, in our analysis of the simulated reads from MetaCyc, we found that as the sequence identity threshold increased, there was an increased fraction of reads that mapped to incorrect nodes. These mapping errors occurred due to ambiguity in the database regarding the reaction catalyzed by the sequences in question: at high identity cutoffs, such ambiguity is often missed (Additional file 1: Figure S1B). Again, the choice of an 80% threshold represents a reasonable compromise in this context.

In our previous work [26], we found that our mapping approach resulted in a set of mapped enzymes that were not obviously deficient in any major enzyme class from MetaCyc. In this analysis, we found that 2767 of 6140 (45%) of all MetaCyc reactions from any known environment were identified at least once in the read data. A list of the top 43 reactions in terms of reads mapped across the 16 animals is presented as Additional file 1: Table S3.

### VFA Analysis

Volatile fatty acids (VFAs) concentrations were ascertained from the rumen fluid samples by centrifuging 5 ml of sample for 10 minutes at 3000 g. The resulting supernatant was then added to a 25% metaphosphoric acid solution containing 2-ethyl butyric acid (2.0 mg/mL). The final ratio of rumen fluid to metaphosphoric acid solution was 5:1. We then incubated the samples for 30 minutes on ice and centrifuged for 30 minutes. The resulting supernatant samples were then added to 1 mL vials for analysis by gas liquid chromatography. VFA concentrations were determined with an Agilent 6890 gas chromatograph following standard procedures. Animal 1127 was absent from our data for technical reasons. The Spearman’s correlation of normalized read counts (from 79, 12, and 7 nodes, for acetate, propionate and butyrate, respectively) and VFA concentrations was computed in R [50].

### Residual feed intake measures

The 16 animals sequenced were selected as they showed extremes in the efficiency with which they converted nutrients into body mass, e.g., their residual feed intake [18]. Thus, RFI was calculated as the deviation of true feed intake from expected feed intake. Expected feed intake was determined by regressing actual feed intake on daily gain and metabolic midweight [51].

### Network Interface

In order to define the interface of the two networks, we created three sets of compounds (hereafter “interface metabolites”) potentially transferred from the rumen microbes to the host. The first set (VFA) consists of the three most abundant ruminal VFAs (>95%): acetate, propionate, and butyrate [52]. The second interface set adds the 20 universal amino acids to the VFAs (VFA + AA). Finally, the third set consisted of VFA + AA plus a set of metabolites known to be absorbed from the gut into cells in the digestive tract of humans. This set, hereafter “ALL,” includes a total of 204 compounds, the full list of which is available as supplemental data from our previous work [26]. Edges between the host and microbial metabolic network join one node in each network that each use an interface metabolite (interface metabolites were considered even if they met the definition of a currency metabolite). See Taxis et al., [26] for further details.

### Layering of Metabolic Networks

We used the *O(n* ^*2*^ *)* algorithm due to Dijkstra [53] to find the shortest path between all pairs of the *n* nodes in the network. For a given microbial (or host) node, the distance from that node to the host (or microbial) network is the length of the shortest path between it and the closest node in the other network. These distances partition the network into layers, where the central layers consist of reactions using interface metabolites (Fig. 1).

### Node read density analysis

Each reaction (node) with at least one mapped read pair was analyzed by using the two-sample Wilcoxon test to test null hypothesis of no difference in normalized read count for the two diets (FORG or CONC), to which we applied a 5% false-discovery rate correction [54].

To assess whether the two diets differed in how reads were distributed among nodes, we fit a three state distribution to the normalized number of reads mapped to each node. The distribution had one proportion of nodes with no reads mapped, a second with 1 read mapped and a third proportion where the number of reads mapped followed a log-normal distribution. We first individually fit this distribution to the read counts from each diet separately. We then compared the sum of the log-likelihoods from this approach to the log-likelihood of fitting the combined read count data to a single distribution of the same form.

### Network structure analyses

We calculated the Pearson correlation between the mapped read counts and network layer for all combinations of networks and interface metabolite sets. We also determined the correlation for the proportion of differentially abundant enzyme genes for each layer (Additional file 1: Table S1).

We next calculated the mean network layer for the mapped reads for each of the two diets across the interface metabolite sets and networks (e.g., N_25_, N_50_, and N_100_; Table 2). To assess whether this mean was significantly different for the two diets, we adopted a randomization approach. First, we pooled the mapped reads across the two diets. Then, we selected at random from these pooled reads the same number of reads as had originally been mapped to the eight FORG animals. We then calculated the difference in mean layer number for the pseudo-concentrate and pseudo-forage reads for each of 1000 randomizations.

**Table 2:** Diet and network position

Group^a^	Currency Cutoff^b^	Mean layer: FORG^c^	Mean layer: CONC^c^	Real Diff.: mean layers^d^	Max. Random difference^e^	*P* ^f^
VFA	N_25_	1.99	1.82	0.17	0.006	**<0.001**
	N_50_	1.87	1.74	0.13	0.006	**<0.001**
	N_100_	1.78	1.64	0.14	0.005	**<0.001**
VFA_AA	N_25_	0.828	0.66	0.16	0.003	**<0.001**
	N_50_	0.87	0.75	0.12	0.004	**<0.001**
	N_100_	0.91	0.80	0.11	0.004	**<0.001**
ALL	N_25_	0.51	0.45	0.06	0.002	**<0.001**
	N_50_	0.46	0.41	0.04	0.002	**<0.001**
	N_100_	0.50	0.47	0.04	0.001	**<0.001**

^a^Interface metabolite set (Materials and Methods)

^b^Network (e.g., currency cutoff; Materials and Methods)

^c^Mean layer number for the reads mapped from FORG or CONC animals, respectively.

^d^Difference between the mean layer for FORG and CONC

^e^Maximum difference in the mean layer for the two diets seen when reads were randomized between the diets

^f^ *P*-value for the test of the hypothesis that the two diets do not differ in mean layer. For this test, reads were randomly reassigned to diets and the mean layers recomputed 1000 times (Materials and Methods *)*. Values significant at *P* = 0.05 shown in bold.

We also analyzed the effect of diet on the metabolic network structure using four statistics:

- “Carbon sum,” the total number of carbon atoms appearing in the reaction associated with that node,
- Betweenness-centrality, namely the total number of shortest paths between any pair of network nodes that pass through the selected node [55],
- Node degree, or the node’s total edge count, and
- Clustering coefficient [56], which indicates the degree to which nodes are clustered in the network.

For each node, we weighted each statistic’s value by that node’s normalized read count and computed the difference in the mean of this weighted value across all nodes between the two diets. We assessed the significance of the differences with the randomization approach above (Additional file 1: Table S2).

### Principal Components Analysis

Principal Component Analysis (PCA) was performed with R [50] using the normalized read counts or the OTU counts previously computed and reported in Ellison et al., [18] as measurements and the 16 animals as experiments.

### Pairwise distances between samples in OTU and node distribution

For each sampled animal, we defined an OTU distribution vector and a node distribution vector *v*, the elements of which are defined as:

1 $$ {v}_i=\frac{r_i}{\sqrt{{\displaystyle {\sum}_{j=0}^n}{\left({r}_j\right)}^2}} $$

where *r*_*i*_ is the number of reads mapped to node/OTU *i* and the denominator scales the resulting vector to unit length. We then computed standard Euclidian distances between all pairs of vectors *v*_*i*_ and *v*_*j*_ from the animals *i,j* for nodes and for OTUs.

We compared these differences to the results of pooling all reads from each diet and randomly reassigning them proportionally to the animals. From these resamplings, we also computed the Pearson’s correlation between node and OTU distances. These randomized correlation values are occasionally high, mostly likely because differences in the number of mapped reads between animals generates outliers. Ordinarily, a non-parametric Spearman correlation would be a more conservative choice for data that are not self-evidently normal. However, because we were comparing the Pearson correlation observed from the real data to those seen in the randomized data, our approach should not suffer from violations of normality assumptions.

### Simulation of a diet shift

The initial diet of the sixteen animals studied was similar to the later FORG diet. We thus sought to assess if the increased node-level variation seen in the concentrate-fed animals (Fig. 3D) might be explicable in terms of this shift. To do so, for each node *n*_*i*_, we defined the set of *k* metabolites participating in that node’s reaction *C(n*_*i*_ *) = {c*_*1*_ *,c*_*2.*_ *…c*_*k*_ *}.* The set of metabolites *C* found in the network as a whole is then simply *C* = *C*(*n*_1_) ∪ *C*(*n*_2_) ∪ *C*(*n*_3_) … ∪ *C*(*n*_*n*_). Here, *C* has 4878 compounds. We define the *i* ^*th*^ element of this compound vector *v* ^*c*^ as follows:

2 $$ {v}_i^c={\displaystyle {\sum}_{j=0}^n\left\{\begin{array}{c}\hfill {\displaystyle {\sum}_{k=1}^8{r}_{j, k}}\hfill \\ {}\hfill 0\hfill \end{array}\right.\begin{array}{c}\hfill {c}_i\in C\left({n}_j\right)\hfill \\ {}\hfill otherwise\hfill \end{array}} $$

where *r*_*j,k*_ is the number of mapped reads for the *j* ^*th*^ node for the 8 CONC animals. For comparative and computational purposes, we then rescale the vector as follows:

3 $$ {V}_i^c=100,000\frac{v_i^c}{{\displaystyle {\sum}_j}{v}_j^c} $$

We defined this scaled average compound vector *V* ^*C*^ as the target. Then we used each FORG animal’s individual compound vector *V* ^*c,i*^ (again computed and scaled as in *2* and *3*) as a starting point. Using simulated annealing [57], we sought a distribution of nodes onto reads that resulted in a compound vector as similar as possible to *V* ^*C*^ *.* Simulated annealing works by taking a starting solution and repeatedly moving to nearby points in the state space in search of better solutions. We used two forms of perturbation. In the first, *swap1*, we simply randomly moved reads between nodes and evaluated whether the new distribution was closer to *V* ^*C*^ *.* In the second, *swap2*, we moved pairs of reads from nodes connected by edges. For each of the eight forage animals, we ran ten simulated annealing analyses for the shift to the concentrate diet and ten for remaining on the forage diet. For each set, we retained the simulation closest to *V* ^*C*^.

## Results

### Metagenomic sequencing of rumen fluid from 16 sheep

We extracted microbial DNA from the rumen fluid from sixteen sheep and then shotgun sequenced those DNA on an Illumina GenomeAnalyzer II, yielding 100 base pair, paired end reads. These reads were not significantly contaminated with host DNA (Table 1). In our previous work, we collectively identified 349 operational taxonomic units (OTUs) from known bacterial and archeal taxa in the rumens of these animals [18]: here we explore the metabolic reactions encoded by these metagenomic samples, using the MetaCyc database [35] as a reference.

### Metabolic network inference

Using MetaCyc, we defined a reaction-centric metabolic network where enzyme-catalyzed reactions are nodes, and any two nodes that share a metabolite are connected by an edge (left side of Fig. 1). Currency metabolites, such as water or ATP, were excluded from the network construction at three stringencies, from high to low: networks N_25_, N_50_ and N_100_, respectively (Materials and Methods). We inferred a metabolic network for the ovine host using the existing human and bovine metabolic networks in MetaCyc via orthology (Materials and Methods). Approximately 8.9 million translated metagenomic read pairs were then mapped to the merged metabolic network (Fig. 1 and Table 1).

### Animal VFA concentration and metabolic network structure have some association

**Fig. 2:**
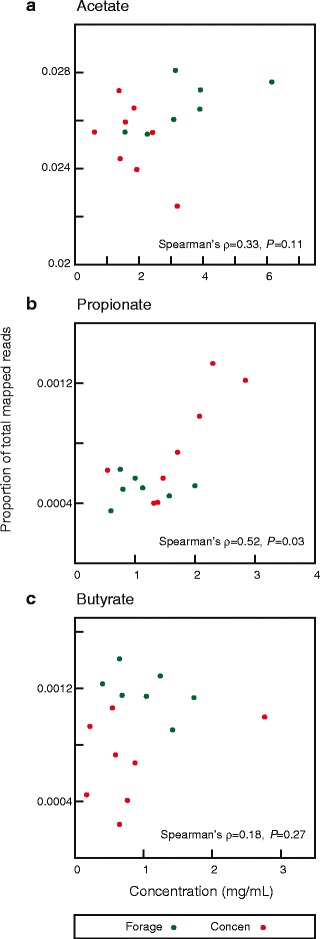
Association of the concentrations of three volatile fatty acids (VFAs) and of reads mapping to reactions involving them. On *x* is the concentration (mg/ml) of the VFA, on *y* is the fraction of reads mapping to reactions using that VFA relative to the total number of reads mapping uniquely to any reaction (e.g., the proportion of all mapped reads that involve that VFA). (**a**) Acetate, (**b**) Propionate, (**c**) Butyrate

Volatile fatty acids (VFAs) make up approximately 70% of dietary energy in sheep [4, 52]. We were curious if the patterns of shotgun sequence data recapitulated the measured VFA concentrations, which might be expected under the hypothesis that organisms encoding enzymes that metabolize particular compounds would occur in the ecosystem in proportion to the abundance of those compounds. Encouragingly, if unsurprisingly, acetate, the most common VFA in the rumen [3], was also a product or reactant of reactions with more reads mapped than any other VFA. A significant correlation between read count and concentration was seen for propionate (Spearman’s ρ = 0.518; *P* = 0.025; Fig. 2), but not for acetate or butyrate (Spearman’s ρ = 0.332, *P* = 0.11; and Spearman’s ρ = 0.175, *P =* 0.27; Fig. 2 *;* respectively). The lack of association between read count and acetate may be because acetate is also key metabolic intermediate and hence many reactions internal to the microbial cells involve it. Butyrate, on the other hand, is the rarest of the three metabolites both in concentration and in mapped reads, making detecting associations difficult.

### Metabolic network structure varies by diet

The two diets differed in the relative density of reads mapped to each node. We fit log-normal distributions to the number of reads mapped to each node (Materials and Methods). There is a significant difference in the distribution mapped to the forage-fed (FORG) and concentrate-fed (CONC) animals (*P <* 10^-10^, likelihood ratio test with 5 degrees of freedom): more reads are mapped to each node in the CONC network than the FORG one (53.8 reads per node per 10^6^ reads mapped and 41.4 reads per node for 10^6^ reads, respectively), with less overall spread in the number of reads mapped per node in the CONC animals (log-variance 2.7 and 2.9, respectively). Similarly, the per-node variation in reads mapped within a diet group is less than that between diets for almost all nodes, reinforcing the point that the two diets differ in their network structure (Additional file 1: Figure S2).

Figure 1B and C illustrate that the FORG animals showed less animal-to-animal variation in the normalized read count for each node than did CONC animals (Mann-Whitney-Wilcoxon test, *P* < 10^-10^). However, fewer total reads were mapped in the CONC animals, so it is possible that this difference in variation is due sample size. We thus randomly drew 100,000 mapped reads for each animal and recomputed the Mann-Whitney-Wilcoxon test on 1000 of these resampled datasets. In all cases, the concentrate-fed animals still showed significantly greater variance (*P <* 10^-9^).

To link the host and microbial metabolic networks, we defined three sets of exchangeable *interface* metabolites (VFA, VFA + AA, and ALL; Materials and Methods). The VFA set contains the three volatile fatty acids that are ruminants’ primary energy source, VFA + AA adds to these the twenty amino acids; while ALL is a large set of metabolites defined by human cellular metabolism (Materials and Methods). Host and microbial network nodes were connected by shared interface metabolites (Fig. 1). We then sorted the merged metabolic network based on each node’s distance to the other subnetwork, resulting in the layered structure of Fig. 1. Intuitively, reactions that involve interface metabolites are found in the innermost layer of each subnetwork (distance 0 from the other subnetwork): they exchange compounds with the other network.

For the two larger sets of interface metabolites (VFA + AA and ALL) there was a general (though not invariable) negative correlation between the number of reads mapped to a reaction and that reaction’s distance from the host subnetwork (Additional file 1: Table S1), similar to the pattern previously seen [26]. There was no significant evidence that the proportion of nodes that differ between the diets varies by layer (Additional file 1: Table S1). However, for the FORG animals, the average read falls into a more distant layer than does the average read for CONC animals (*P <* 0.001; Table 2), meaning that the FORG reads map more often to reactions distant from the host network than do reads from the CONC samples. This difference in mean layer is more than 20 times as large as any difference in mean layer seen among our 1000 randomizations of the reads into the two diets (Table 2).

The network structure induced by the two diets also differs (Additional file 1: Table S2). Reads from the CONC diet mapped more often to nodes with higher degree (e.g., larger number of edges) and higher betweenness-centrality (meaning that these nodes lie on “key paths” through the network; Materials and Methods). CONC reads also mapped to nodes with higher clustering coefficients. On the other hand, the mean number of carbon atoms involved in a node’s reaction was significantly larger for the FORG reads (*P <* 0.001 by network randomization, Additional file 1: Table S2). These results are in accord with the observation that CONC reads generally map to layers closer to the host network than do FORG reads, since these nearer reactions are also more central in the network and hence involve smaller metabolites.

### Diet differences are not driven by reaction presence/absence.

While the two groups of animals showed many differences in the compositions of the microbial metabolic networks, these differences are not primarily driven by the absence of reactions/nodes in one diet group. Of the 2767 nodes observed in either diet, 2254 were observed in both, while only 292 were specific to FORG and 221 to CONC. Moreover, most of these apparent differences can be explained by sampling effects: only 30/292 of the apparent differences for FORG and 47/221 of those for CONC were statistically significant (*P <* 0.01). Instead, most differentially present nodes are simply rare: the mean number of reads mapped to nodes exclusive to FORG and CONC animals were 3.6 and 9.2, respectively, while the maximum number of reads mapped to a differentially present node was 318 (Additional file 1: Figure S3).

### Principal components analysis identifies metabolic variation in CONC animals

**Fig. 3:**
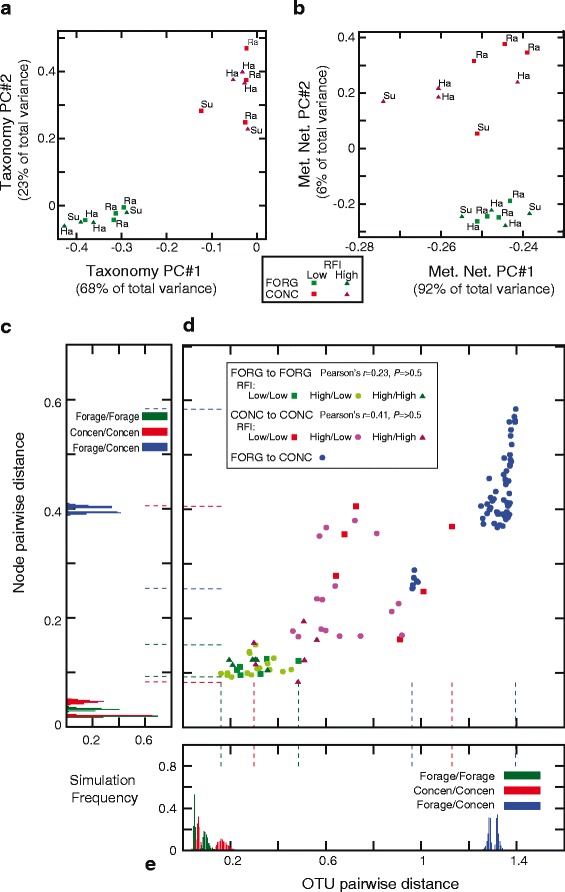
Animal-to-animal taxonomic and network differences. **a** Principal component analysis of the OTU distributions across the 16 animals. The first two principal components (PCs) are shown, comprising 92% of the total variance. FORG animals are shown in green and CONC in red. Visually, it seems clear that the diet difference explains most of the variation in OTU distributions. An animated 3-dimensional version of this plot that includes PC#3 (3% of variance) is presented as Additional file 2. Each point is labeled with the breed of the animal in question: Su: Suffolk, Ra: Rambouillet, Ha: Hampshire. **b** Principal component analysis of the distribution of reads mapped to metabolic network nodes. The first two principal components (PCs) are shown, comprising 98% of the total variance. However, diet is no longer the main source of variation. Instead, principal component 1 separates three CONC animals (numbers 1220, 1239 and 1348; high RFI) from the rest of the dataset. Inspection of the node-level data suggests that these three animals are unusual in that they have higher than usual node-to-node variation in the number of mapped reads (namely a few nodes with a large number of mapped reads) and are also highly correlated with each other, unlike some of the other CONC animals with rather different profiles. An animated 3-dimensional version of this plot that includes PC#3 (1% of variance) is presented as Additional file 3. **c** Minimum and maximum pairwise node distances seen when reads were randomly and proportionally reassigned to each animal. On the *y*-axis is the same distance scale as *y* in panel D, on the *x*-axis is the proportion of simulations with a given minimum/maximum (Materials and Methods). The color scheme is as for (**d**) Dashed lines give the minimums and maximums seen in the real data of **d. d** Pairwise differences in distribution of reads mapped to OTUs (*x*-axis) and nodes (*y*-axis). FORG to FORG comparisons are shown in green, CONC to CONC in red, and FORG to CONC in blue. For each animal, a vector representing all mapped reads was normalized to unit length and then standard Euclidian distances computed between it and all other animals (Materials and Methods). For the FORG to FORG and CONC to CONC pairs, we computed the Pearson’s correlation of OTU and node distance and compared that value to that seen from randomized datasets (Materials and Methods). **e** As for **c**, except with the OTU distances. The *x-*axis gives OTU distances and the *y*-axis simulation frequencies

We used PCA to explore how diet interacts with the animal-to-animal variation in microbial taxa (OTUs) and reactions (nodes). Neither the OTU nor the node-based PCA analyses suggested that the sheep breed was a strong confound to our results (see also Additional file 1: Figure S4), though we note that the small number of animals from each breed in each treatment makes it difficult to clearly observe the effect of this variable on the microbiome. For taxonomy, diet appears to be predominant driver of animal-to-animal differences (PC1 and PC2; Fig. 3A). However, with the metabolic network nodes, most of the variation is accounted for by a PC that separates not diets but three CONC animals from the remaining 13 animals (Fig. 3B, PC1). These three animals were inefficient in their growth relative to the food consumed, e.g., had high residual feed intake or RFI [18], and all three had similar node profiles, with a few nodes with very large numbers of mapped reads and the others having many fewer (Fig. 3B).

To assess if our PCA was overly confounded by assumptions of normality, we also computed principle components using instead Spearman’s and Kendall’s correlation statistics [58]: the results of which are shown in Additional file 1: Figure S4. On the basis of these analyses, we conclude that some aspects of the standard PCA might have been confounded by the non-normal nature of the data. In particular, the role of diet in PC1 for the taxonomy data is dependent on the association measure used. However, in general, the observation that the forage animals are more similar in their metabolic networks than were the concentrate-fed animals was supported by all of these analyses.

### Concentrate-fed animals show large pairwise distances between each other in both taxonomy and in the metabolic network

Given that the principle component analyses were not completely informative, we sought to more explicitly explore this apparent difference between the two diets in variability with a pairwise distance analysis. For both the number of reads mapped to nodes and to OTUs, the FORG animals showed small and relatively uniform pairwise differences, although these differences were still larger than can be explained by sampling (*P <* 0.001). For the CONC animals, there was a much wider spread of distances both for the OTUs and for the nodes (*P <* 0.001; Fig. 3D), although the three CONC animals with high RFI discussed above were quite close to each other. On the other hand, some of the pairwise differences between low RFI (high efficiency) CONC animals were as large as for pairs of animals fed differing diets (Fig. 3D).

### Simulation of a diet shift

**Fig. 4:**
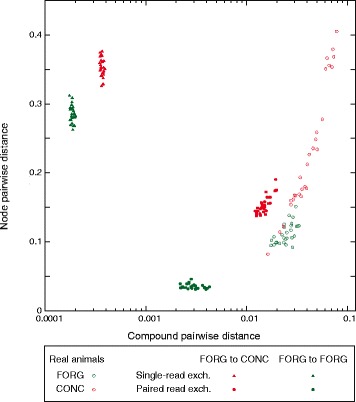
Simulation of a shift in diet. We used simulated annealing to model a shift in diet from FORG to CONC (red hues). The average compound distribution of the eight CONC animals was used as a target and the eight FORG animals as the starting points (Materials and Methods). We used two different sampling schemes in the simulated annealing: Single-read exchanges moved reads from one node to another until the resulting compound vector was minimally different from the target (triangles), while paired read exchanges moved two reads at a time under the constraint that the two nodes that both reads originated from and the two nodes that they moved to had to, in each case, be connected by an edge (circles). Shown in green hues are the parallel simulations that started with FORG animals but used the average FORG compound vector as a target. On the *x-*axis we show the results of pairwise comparisons in compound distance between all combinations the eight simulated animals, using for each its best simulated annealing run. The open points show, for reference, the distribution seen in the real animals. On the *y-*axis are the pairwise node distances for these same comparisons

To explore the effect of diet shift on the metabolic network, we sought to assess whether there might be multiple different metabolic networks (e.g., sets of enzymes) that nonetheless have a very similar distribution of the metabolites used by those enzymes. To do so, we defined a target metabolite vector, which was defined as the average number of reads, for one of the diets, that mapped to any enzyme that employs that metabolite. Using this average vector as a target and each of the eight forage animals as a starting point, we used simulated annealing to search for distributions of reads onto nodes (e.g., enzymes) that produced a mapping of reads onto metabolites that was as near in vector space as possible to the original target metabolite vector. In this framework, if the target vector was from the concentrate diet, we could simulate a diet shift, while if the target were from the forage-fed animals, we would simulate the maintenance of the same diet. For each animal and simulated diet shift, we retained the simulated annealing run (out of ten) nearest to the average compound vector. In Fig. 4, we show the (8 × 7)/2 pairwise comparisons of these simulations both for compound and node distance. We ran the simulations under two types of “move rules.” In the first, we simply moved reads between nodes at random and retained moves according to the simulated annealing selection function [59]. These simulations found node distributions very close to the target compound vector (triangles in Fig. 4). However, real enzymes are linked in the microbiome by genomic co-occurrence and hence cannot change this arbitrarily. While we do not know the genomes from which our nodes derive, we can to some degree simulate this effect by not moving single reads but rather by selecting pairs of connected nodes, and then moving one read from each node to another pair of connected nodes. This second set of simulations (filled circles, Fig. 4) shows differences that are considerably more similar to those of the real animals. What both sets of simulations show, however, is that there exists a range of potential metabolic networks that, despite their compound similarity, are quite different in the set of metabolic reactions that generate those compound distributions.

## Discussion

Not surprisingly, diet drives significant changes in the microbial ecosystem of the rumen. As our previous work suggested at the taxonomic level [18], these differences, under these experimental conditions at least, are not reflected in the loss and gain of metabolic reactions or microbial species, but rather by shifts the relative abundance of those components. Many of the metabolic changes are also to some degree expected: the metabolically simpler CONC diet uses enzymes that more directly connect to host metabolism and to each other (Table 2 and Additional file 1: Table S2) and that employ shorter carbon chains. These observations increase our confidence that this metabolic network-driven approach reflects important underlying patterns in the microbiome. As an aside, we note that reanalyzing the combination of network N_50_ and interface set VFA + AA with a sequence identity cutoff of 90% for read mapping yields the same conclusion of smaller mean layer number and higher variance in the CONC samples (data not shown), meaning that sequence identity is not likely a major confounding factor in our analyses. Similarly, while the correlations between metabolite levels and microbial read counts in the VFA analysis were relatively weak, the fact that any association is evident in such a small sample of animals may be encouraging. We also further illustrated the dangers of assuming a close association between the metabolic and taxonomic patterns in the microbiome [7, 23–28], finding that the pairwise differences in taxonomy and in metabolism for our animals are not strongly associated (Fig. 3C). We do however note that we cannot be certain that sampling limitations may not account for some of this lack of association. Finally, the fact that the taxonomic and metabolic differences seen are differences in abundance and not of presence or absence reinforces the importance of using network modeling and large-scale analyses to analyze these ecosystems. We believe that describing the microbiome primarily at the level of individual pathways can be misleading because such descriptions are necessarily incomplete with respect to these networks.

The more striking observation from our study, however, is that the shift to the CONC diet induced large and variable changes in the microbial metabolic networks of the animals that experienced it. Thus, even though on average the CONC animals had networks that were simpler and closer to the host network, these eight animals were quite distinct from one another in their node distributions (and, to a lesser extent, their taxonomic distribution). This difference in the amount of variation between the diets is evident in the principal component distributions of Fig. 3B and from the pairwise distances in Fig. 3C. It is tempting to ascribe this difference to the fact that the FORG diet is more similar to the evolved diet of these animals. Thus, while foregut fermentation has convergently evolved in herbivores multiple times [60, 61], ruminants represent probably the most complete and dramatic adaptation to allow efficient digestion of fibrous plant diets, and they possess very distinct microbiota [22]. Nonetheless, we believe that an equally likely explanation lies in the structure of our feeding experiment. The animals were all originally on a forage-like diet, so it is possible that it was the switch to the CONC diet that allowed this animal-to-animal variation to arise. Indeed, our simulations (Fig. 4) show that, under rather simple assumptions, it is possible to create divergent metabolic networks (e.g., enzyme abundances) that share very similar metabolite profiles. In keeping with this model, we find that animal-to-animal differences, both between and within diets are driven by abundance differences, not enzyme or taxa presence or absence (Additional file 1: Figure S3). Given the experimental design and these simulations, we propose as a null hypothesis to explain our observations above the idea that diet shifts can alter the microbial ecosystem in non-repeatable ways.

Another observation that supports this idea is that there were three CONC animals that were actually quite similar to each other in their node distributions. Importantly, these were also three of the four animals that appeared to have adapted poorly to the new diet: e.g., they consumed a surprising amount of feed for the amount of weight they gained. With only 4 low and 4 high RFI animals per diet group, we do not wish to overstate the strength of this result. But it seems plausible that one of the outcomes of a diet switch might be a collapse in metabolic system diversity, which can occur in a parallel fashion in different animals and results in an inefficient use of ecosystem resources. This conclusion has a very interesting link to data from humans, where microbiomes having low gene complexity (relatively few identified genes) are associated with several indicators of poor health [29]. Indeed, the question of whether complex and diverse ecosystems are more productive than less diverse ones is an old and incompletely resolved one in ecology: while theoretical and experimental analyses have suggested that this is often the case [62, 63], the mechanisms that would drive this effect; e.g., the presence of rare but beneficial taxa, cooperative interactions between taxa or more efficient filling of ecosystem niches [62, 64], remain unclear.

While many of the arguments on ecosystem functioning are made from a taxonomic diversity perspective, the underlying processes are likely to be due to the functional characteristics of those taxa [62]: in particular their metabolic capacities. The importance of this principle is illustrated by the fact that the metabolic behavior of an ecosystem need not be strongly coupled to its taxonomic structure [28]. As such, there is an enormous need for ecosystem-scale metabolic models. A very promising avenue for developing such models is to build off of the success of existing genome-scale metabolic models [31, 65] by coupling them to metagenomic data using enzyme-mapping tools such as those presented here. At the moment, unfortunately, such models do not scale to more than a handful of species at once [65–68]. However, there do not appear to be fundamental limitations to this approach [31, 34, 65], so that questions such as why degenerate ruminal ecosystems show poor performance may be addressable predictively and quantitatively.

Another open question is whether ecosystems, in addition to being robust to taxonomic variation [28], also allow for the existence of different community metabolic networks [26]. Here, our computational simulations of a diet shift (Fig. 4) show similarities to our observed data in that both have community metabolic networks that are quite distinct from one another, suggesting that a change in ecosystem parameters can open the way for the formation of a number of different community metabolic networks. In fact, despite using a very simple set of rules to govern these simulations, to a degree we were able to replicate pairwise host animal differences in both compound distribution and in node read mapping distributions. To do so, all that was necessary was to impose a rule that reads mapping to pairs of related reactions could only move together. This rather simple rule mimics the fact that reactions do not move in the community metabolic network alone but as part of (unknown) genomes. Our claim thus is not that our simulations define the mechanism of the diet switch but merely that, under some constraints, selection to move the community network to a different compound distribution can produce metagenomic data similar to that actually observed. Thus, the low RFI (efficient) CONC animals all appear to have found different metabolic networks that nonetheless function well, linking again to evolutionary studies that have shown that metabolism is highly flexible and robust, with multiple solutions to many metabolic problems [69, 70]. Thus, in contrast to Tolstoy’s families, it may be that it is unhappy microbiomes that are alike, while each happy microbiome is happy in its own way.

## Conclusion

Metabolic network analyses of the ovine rumen microbiome illustrate how changes in diet alter not merely the taxonomic composition of this environment, but also the balance of metabolic reactions present. A diet richer in simple carbohydrates (a concentrate diet) induces a metabolic network closer in network space to the host’s reactions, while a diet composed of complex plant matter (a forage diet) induces a more complex network that is also more distant from that of the host. The shift from a forage to a concentrate diet also perturbs the metabolic network, resulting in more animal-to-animal variation in individuals fed the latter diet. This pattern of multiple ecosystem-level metabolic networks with similar properties can be partly replicated by network simulations of the process of the diet shift, suggesting that such metabolic networks maintain considerable flexibility.


Additional file 2
Additional file 3


## Additional files

Additional file 1: Supplemental Figures 1-3 with captions; Supplemental Tables 1-3. (PDF 6395 kb)

## Abbreviations

CONC: Concentrate-based diet
FORG: Forage-based diet
OTU: Operational taxonomic unit
PCA: principal component analysis
RFI: residual feed intake
