# Supplementary material for: Diet shifts provoke complex and variable changes in the metabolic networks of the ruminal microbiome
Source: Microbiome. 2017 Jun 8;5:60. doi: 10.1186/s40168-017-0274-6 (PMC5465553; doi:10.1186/s40168-017-0274-6)
Supplement: Supplementary file 3 — Supplemental Figures 1-3 with captions; Supplemental Tables 1-3. (PDF 6395 kb) [file 40168_2017_274_MOESM1_ESM.pdf]

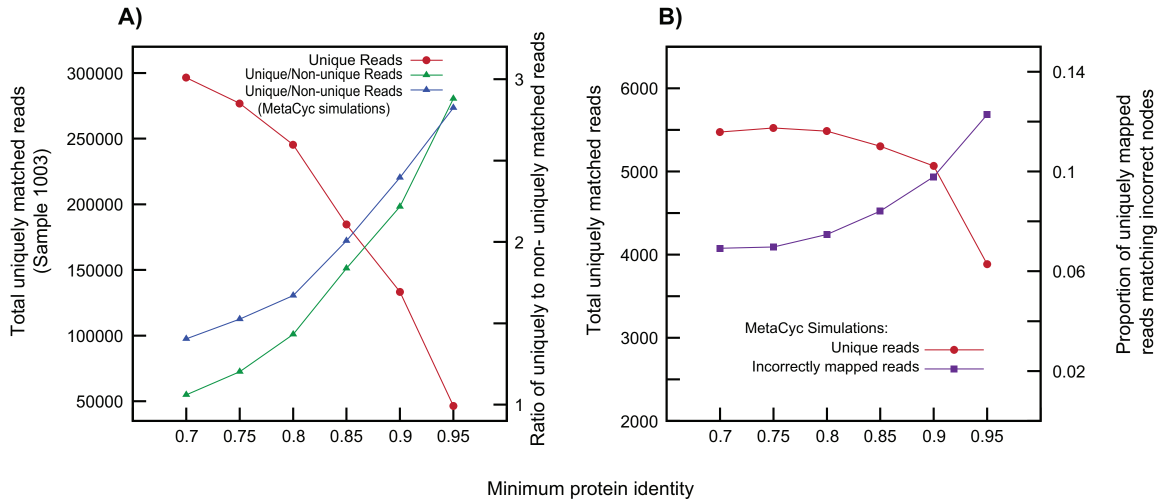

Supplemental Figure 1. Sequence identity thresholds and mapping results. A) Effect of differing minimum sequence identity thresholds between translated reads and the enzyme database on the total number of uniquely matched reads (left axis) and on the relative proportion of matched reads not matching to a unique reaction (right). On the *x*-axis is the minimum protein sequence identity (for both members of a read pair) required to define a match to a sequence in the enzyme database (Sample 1003 was considered). On the *y*-axis to the left is the total number of reads matching a unique reaction from this sample (from the total of 14,521,805 reads in this sample that passed quality filtering and minimum ORF length thresholds; see *Materials and Methods* and Table 1). On the right *y*-axis is the ratio of the number of reads mapping to unique reactions over the number of reads matching enzyme sequences catalyzing more than one distinct reaction. Hence, as this ratio approaches 1.0, more reads that match the database cannot be uniquely assigned to a reaction. In green are these statistics from the analysis of sample 1003. In blue are the same statistics computed from 20,000 simulated reads created from the MetaCyc database (see *Materials and Methods*). Below 80% identity, the number of new reads matched drops off, while the number of non-uniquely matched reads approaches the number of uniquely matched ones, partly motivating our selection of 80% identity as our cutoff (*Materials and Methods)*. Somewhat surprisingly, the ratio of unique to non-uniquely mapping reads is quite similar for the real data from sample 1003 and for simulated reads created from the MetaCyc sequences. **B)** Behavior of the mapping approach on simulated reads from MetaCyc. Using the 20,000 simulated reads from **A** (with lengths and insert sizes similar to our real data), we counted the number of such simulated reads that uniquely mapped to nodes (red curve, left axis). Because we omitted from matching the parent sequence used to create each simulated read, between 50% and 72% of simulated reads found no match in the database (for 70% to 95% identity cutoffs, respectively). Hence, the number of reads uniquely matching nodes decreases as the identity threshold increases. We also computed how often a read was uniquely mapped to a node other than the node corresponding to the sequence it was simulated from (purple curve, right axis). Such an error in mapping can occur when sequences that are similar nonetheless catalyze differing reactions, or when annotations fail to account for multiple enzymatic activities for a single enzyme. As can be seen, at high identity thresholds, these errors are more common, because there is less chance that the read in question will match to a second sequence with a distinct reaction. Hence, by using a lower sequence identity threshold, we will tend to categorize more reads into the set of non-unique mappings (right axis of **A**) and make fewer positive assignment errors of reads to the wrong reaction. Note that these percentages most likely overstate the error rate from our real data as we were forced to omit matches from the simulated reads corresponding to the sequence from which they were simulated. Since these simulated reads generating errors derive from enzymes having a near sequence neighbor with a different activity, when we omit the generating sequence, the database loses information on this conflict. In the actual analyses, such conflicting reads are not omitted and there is less chance of incorrectly assigning a read to a single node that is incorrect.

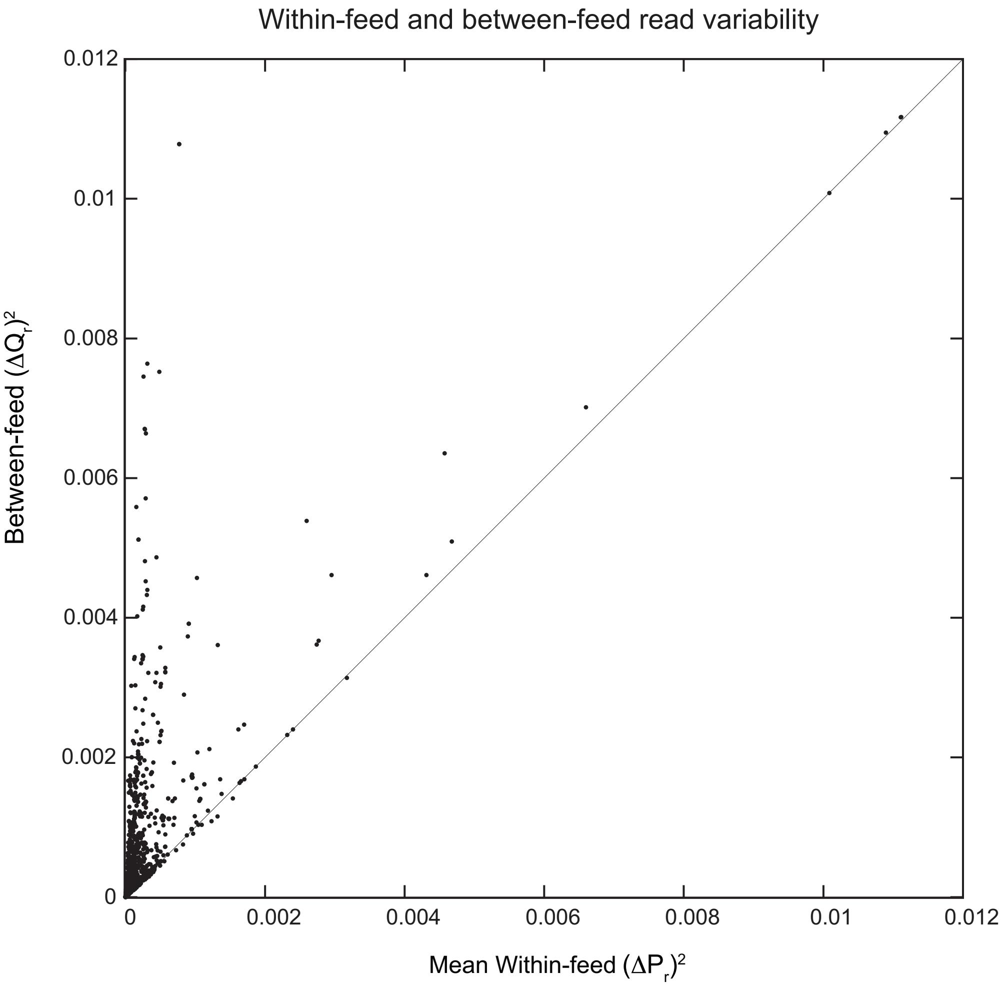

Supplemental Figure 2. Within-feed animal to animal variability is less than the variability between the two feeds. For each metabolic network node, we calculated P_r for each animal: the proportion of the total mapped reads assigned to that node. We next calculated the variability between animals fed the same diet (*x*-axis) as:

$$(\Delta P_r)^2 = \sum_{i=1}^{n} \sum_{j=i+1}^{n} \left(P_i - P_j\right)^2 \Big/ \left(\frac{\sum_{i=0}^{m} P_i}{m} \cdot \frac{n(n-1)}{2}\right)$$

Where *n* is the number of animals in a particular feed-group and *m* is the total number of animals. The quantity plotted is thus the mean squared difference between animals in the proportion of reads mapped to that node for a particular feed, divided by the mean proportion of reads mapped to that node across both feeds. We averaged this value for the two feeds to obtain the values on the *x*-axis above. Similarly, we calculated the mean variability between animals fed different diets for the *y*-axis:

$$(\Delta Q_r)^2 = \sum_{i=0}^{n} \sum_{j=0}^{n} \left(P_i - Q_j\right)^2 \Big/ \left(\frac{\sum_{i=0}^{m} P_i}{m} \cdot n^2\right)$$

Where P_i is the proportion of reads mapped in animal *i* from the first feed and Q_j is the proportion of

animals mapped in animal *j* of the second group. Thus, $(\Delta Q_r)^2$ is the variability in proportion of mapped reads between the two feeds, normalized by the average proportion of reads mapped for that node. A line of y=x is shown for reference: as is clear, it is almost invariably the case that there is more variability between the two diets than within a diet, as would be expected if the two diets differ in the types of enzymes needed to digest them.

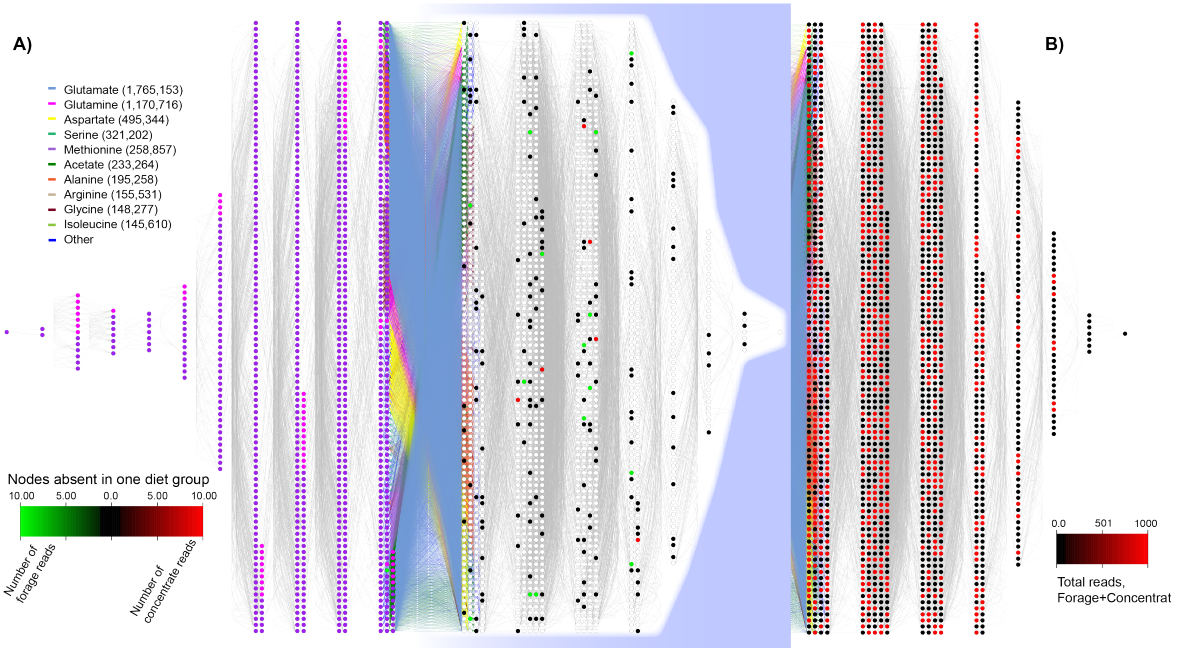

Supplemental Figure 3. Node presence/absence does not explain the differences between the FORG and CONC diets. **A)** Shown are all of the nodes present in FORG and absence in CONC (green) or vice-versa (red). The color intensity is proportion to the total number of reads seen in the diet where the node is present. Note relatively low number of reads that result in a fully colored node (lower left). The maximum of reads mapped to any node in this panel is 318. **B)** For comparison, the same network topology with the total number of reads mapped across both diets for all nodes. Note the much greater range of read counts in this version of the network (lower right).

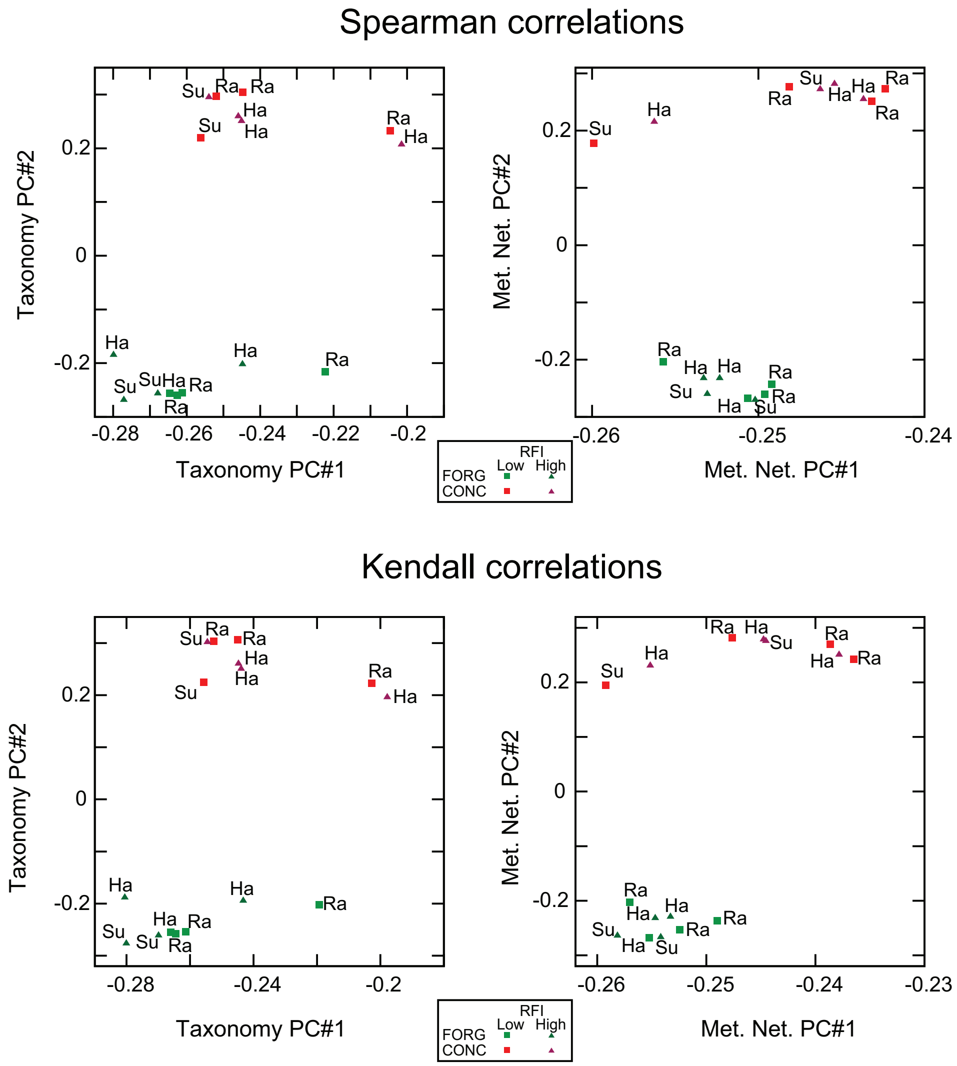

Supplemental Figure 4. Principal component analysis of the OTU and node distributions across the 16 animals using differing correlation statistics. The first two principal components (PCs) are shown in all cases. FORG animals are shown in green and CONC in red. Upper group: PCs computed using Spearman correlations, lower group: PCs computed with Kendall correlations. Each point is labeled with the breed of the animal in question: Su: Suffolk, Ra: Rambouillet, Ha: Hampshire.

Supplemental Table 1. Read count/Network Level Correlation

		Read count vs. level (FORG)		Read count vs level (CONC)		Proportion of differential reads and level	
**Group**[a]	**Currency Cutoff**[b]	**Pearson's *r***[c]	**P-value**[d]	**Pearson's *r***[c]	**P-value**[d]	**Pearson's *r***[e]	**P-value**[d]
VFA	N_{25}	0.230	0.576	0.219	0.549	**0.635**	**0.033**
	N_{50}	-0.166	0.105	-0.200	0.085	-0.197	0.700
	N_{100}	-0.199	0.068	**-0.276**	**0.035**	-0.221	0.324
VFA_AA	N_{25}	**-0.461**	**0.012**	**-0.619**	**0.003**	0.060	0.413
	N_{50}	**-0.255**	**0.006**	**-0.446**	**0.000**	0.287	0.325
	N_{100}	**-0.281**	**0.006**	**-0.461**	**0.000**	-0.039	0.322
ALL	N_{25}	0.168	0.364	-0.037	0.162	0.465	0.222
	N_{50}	**-0.506**	**0.002**	**-0.595**	**0.000**	-0.476	0.604
	N_{100}	**-0.425**	**0.017**	**-0.580**	**0.001**	-0.620	0.817

a: Interface metabolite set (*S1*)
b: Network (e.g., currency cutoff; *S1*)
c: Correlation of the number of reads mapped to a layer and that layer's distance to the host network (i.e., negative correlations imply fewer reads mapped to layers more distant from the host). Calculated for FORG and CONC, respectively.
d: *P*-value for the test of the hypothesis that the correlation in read count or in differential reads is larger than can be explained by chance; assessed by randomization of layer numbers with respect to read counts. Bold values are significant at P=0.05.
e: Correlation of the proportion of nodes with differential read abundance (*S1*) between FORG and CONC animals verses the nodes' layer number.

Supplemental Table 2. Network structure and diet

**Statistic**[a]	**Network**[b]	**FORG**[c]	**CONC**[c]	**Real Difference (FORG-CONC)**[d]	**Mean Random Difference**[e]	***P***[f]
Carbon Sum	N_{25}	33.225	32.438	0.787	0.002	**< 0.001**
	N_{50}	33.225	32.438	0.787	0.001	**< 0.001**
	N_{100}	33.225	32.438	0.787	0.001	**< 0.001**
Betweenness Centrality	N_{25}	9118.7	9423.4	304.7	1.069	**< 0.001**
	N_{50}	18318.2	20949.1	2630.9	2.148	**< 0.001**
	N_{100}	18542.9	22280.6	3737.6	1.100	**< 0.001**
Degree	N_{25}	3.544	3.622	0.078	<0.001	**< 0.001**
	N_{50}	9.172	9.316	0.143	<0.001	**< 0.001**
	N_{100}	12.154	12.984	0.830	0.001	**< 0.001**
Clustering Coefficient	N_{25}	0.882	0.886	0.004	<0.001	**< 0.001**
	N_{50}	0.859	0.862	0.003	<0.001	**< 0.001**
	N_{100}	0.846	0.847	0.001	<0.001	**< 0.001**

a: Network statistic compared between diet (*S1*). Carbon sum: Total number of carbon atoms involved in a reaction. Betweenness-centrality: Number of shortest paths crossing through a node. Degree: Number of edges of a node. Clustering coefficient: Number of fully connected triangles a node participates in over the total number of edges.
b: Network (e.g., currency cutoff; *S1*)
c: Mean value per read for the selected network statistic for FORG or CONC animals, respectively.
d: Difference between the mean statistic value for FORG and CONC
e: Mean difference between the two diets when reads are randomly assigned to the two diets.
f: *P*-value for the test of the hypothesis that the two diets do not differ in mean statistic. For this test, reads were randomly reassigned to diets and the network statistics recomputed 1000 times (*S1*). Values significant at *P*=0.05 shown in bold.

Supplemental Table 3—Most common nodes and their corresponding reaction

Node[a]	Reaction[b]
Node1815_RXN0-5248	H^+ + NAD^+ + E- --> NADH
Node5564_GLUTAMINESYN-RXN	Ammonium + L-Glutamate + ATP + Ammonia --> *Pi* + H^+ + ADP + L-Glutamine
Node2965_ISOLEUCINE--TRNA-LIGASE-RXN	*L-Isoleucine-tRNAs* + H^+ + L-Isoleucine + ATP --> *Charged-L-Isoleucine-tRNAs* + PPI + AMP
Node1249_ASPARTATEKIN-RXN	L-Aspartate + ATP --> L-Beta-Aspartyl-P + ADP + H^+
Node786_GLYMALTOPHOSPHORYL-RXN	*Pi* + *Glycogens* --> Glucose-1-Phosphate + Maltotetraose
Node4498_VALINE--TRNA-LIGASE-RXN	*Valine-tRNAs* + H^+ + Valine +ATP --> PPI + *Charged-VALINE-tRNAs* + AMP
Node2958_GLUTAMATE-SYNTHASE-FERREDOXIN-RXN	*Oxidized-ferredoxins* + L-Glutamate --> *Reduced-ferredoxins* + H^+ + 2-Ketoglutarate + L-Glutamine
Node3747_PYRUVATEORTHOPHOSPHATE-DIKINASE-RXN	Pyruvate + *Pi* + ATP --> PPI + Phospho-Enol-Pyruvate + H^+ + AMP
Node5588_ACETYLORNDEACET-RXN	N-Alpha-Acetylornithine + Water --> Acetate + L-Ornithine
Node6014_GLUTAMIN-RXN	Water + L-Glutamine --> Ammonium + H^+ + L-Glutamate + Ammonia
Node5614_NADH-DEHYDROG-A-RXN	Ubiquinone-8 + H^+ + NADH + *Ubiquinones* --> *Ubiquinols* + H^+ + Ubiquinol-8 + NAD
Node1684_5.99.1.2-RXN	*Negatively-super-coiled-DNAs* --> *Relaxed-DNAs*
Node1361_GLUTAMATE-SYNTHASE-NADH-RXN	L-Glutamate + NAD --> H^+ + 2-Ketoglutarate + L-Glutamine +NADH
Node1615_LEUCINE--TRNA-LIGASE-RXN	H^+ + *Leucine-tRNAs* + Leucine + ATP --> *Charged-Leucine-tRNAs* + PPI + AMP
Node1402_RXN0-4261	*Supercoiled-Duplex-DNAs* + ATP --> *Pi* + ADP + *Single-Stranded-DNAs*
Node1825_PEPCARBOXYKIN-RXN	Oxalacetic Acid + ATP --> Phospho-Enol-Pyruvate + Carbon Dioxide + ADP + H^+
Node3276_RXN-1826	*Pi* + *Long-linear-glucans* --> Glucose-1-Phosphate + *Long-linear-glucans*
Node5835_PHENYLALANINE—	*Phenylalanine-tRNAs* + H^+ + Phenylalanine + ATP --> PPI + AMP

TRNA-LIGASE-RXN	+ *Charged-Phenylalanine-tRNAs*
Node880_RXN0-5182	*Pi* + Maltotetraose --> Glucose-1-Phosphate + Maltotriose
Node3080_ALANINE--TRNA-LIGASE-RXN	H^+ + *Alanine-tRNAs* + L-Alpha-Alanine + ATP --> PPI + *Charged-Alanine-tRNAs* + AMP
Node2708_DNA-DIRECTED-RNA-POLYMERASE-RXN	RNA-N + *Ribonucleoside-Triphosphates* + *Nucleoside-Triphosphates* + *RNA-Holder* + *RNAs* --> PPI + RNA-N + *RNA-Holder* + *RNAs*
Node1996_3.6.5.1-RXN	GTP + Water --> *Pi* + H^+ + GDP
Node5263_4.1.1.32-RXN	GTP + Oxalacetic Acid --> Phospho-Enol-Pyruvate + Carbon Dioxide + GDP
Node3818_CELLOBIOSE-PHOSPHORYLASE-RXN	*Pi* + Cellobiose --> Glucose-1-Phosphate + Glucose
Node581_GLUTAMATESYN-RXN	NADP + L-Glutamate --> H^+ + 2-Ketoglutarate + L-Glutamine + NADPH
Node400_RXN-12392	*Pi* + *Linear-Malto-Oligosaccharides* --> Glucose-1-Phosphate + *Linear-Malto-Oligosaccharides*
Node3560_RXN0-5184	*1-4-alpha-D-Glucan* + *Pi* + Amylosen --> *1-4-alpha-D-Glucan* + Glucose-1-Phosphate + amylosen-1
Node5731_RXN-13519	H^+ --> H^+
Node5849_RXN0-5330	H^+ + NADH + *Ubiquinones* --> *Ubiquinols*+ NAD^+
Node4144_GLUTDEHYD-RXN	Water + NADP + L-Glutamate --> H^+ + Ammonium + 2-Ketoglutarate + NADPH + Ammonia
Node5917_RXN-12171	*Maltodextrins* + *Pi* --> *Maltodextrins* + Glucose-1-Phosphate
Node4246_METHIONINE--TRNA-LIGASE-RXN	H^+ + Methionine + *Methionine-tRNAs* + ATP --> PPI + AMP + *Charged-Methionine-tRNAs*
Node4952_3.6.3.14-RXN	Water + H^+ + ATP --> *Pi* + ADP + H^+
Node2493_METHYLMALONYL-COA-MUT-RXN	Methyl-Malonyl-CoA --> Succinyl-CoA
Node3482_PEPSYNTH-RXN	Water + Pyruvate + ATP --> *Pi* + Phospho-Enol-Pyruvate + H^+ + AMP
Node652_FGAMSYN-RXN	Water + 5-P-Ribosyl-N-Formylglycineamide + L-Glutamine + ATP --> 5-Phosphoribosyl-N-Formylglycineamidine + *Pi* + H^+ + ADP + L-Glutamate
Node1263_RXN-12481	ITP + Oxalacetic Acid --> IDP + Phospho-Enol-Pyruvate + Carbon Dioxide
Node4507_THREONINE--TRNA-LIGASE-RXN	Threonine + H^+ + *Threonine-tRNAs* + ATP --> PPI + AMP + *Charged-Threonine-tRNAs*

Node476_4.1.1.38-RXN	PPI + Oxalacetic Acid --> *Pi* + Phospho-Enol-Pyruvate + Carbon Dioxide
Node4554_GLYCOPHOSPHORYL-RXN	Pi + *Glycogens* --> CPD0-971 + Glucose-1-Phosphate + CPD-1790
Node1581_5.99.1.3-RXN	*Double-Stranded-DNAs* + *Supercoiled-Duplex-DNAs* + *Relaxed-DNAs* + ATP --> *Pi* + ADP + *Negatively-super-coiled-DNAs* + *Relaxed-DNAs*
Node4132_RXN0-5244	H^+ + *Ubiquinones* + E- --> *Ubiquinols*
Node2277_RXN-12878	Water + *Oxidized-ferredoxins* + L-Glutamate --> *Reduced-ferredoxins* + H^+ + Ammonium + 2-Ketoglutarate + Ammonia

a: Node name in our naming scheme
b: Reaction associated with that node
